# Efficacy of Momelotinib in Myelofibrosis Patients: Results From a Multicenter Study

**DOI:** 10.1111/ejh.70034

**Published:** 2025-09-16

**Authors:** Maria Carmen Martorelli, Novella Pugliese, Maria Di Perna, Danilo De Novellis, Assunta Lombardi, Laura De Fazio, Giuseppina Loglisci, Ilaria Peluso, Matteo Molica, Carolina Copia, Alessandra Ricco, Antonella Bruzzese, Francesco Mendicino, Vincenza De Fazio, Teresa Maria Santeramo, Rosario Bianco, Giuseppe Monaco, Raffaele Fontana, Roberto Guariglia, Bianca Serio, Serena Luponio, Anna Maria Della Corte, Antonella Malandrini, Valentina Giudice, Ferdinando Frigeri, Giuseppe Tarantini, Attilio Guarini, Massimo Gentile, Pellegrino Musto, Mario Annunziata, Nicola Di Renzo, Marco Rossi, Catello Califano, Fabrizio Pane, Carmine Selleri

**Affiliations:** ^1^ Hematology and Transplant Center University Hospital “San Giovanni di Dio e Ruggi d'Aragona” Salerno Italy; ^2^ Hematology and Hematopoietic Stem Cell Transplant Center University of Naples Federico II Naples Italy; ^3^ Hematology Unit Hospital “Andrea Tortora” Pagani Italy; ^4^ Department of Medicine, Surgery, and Dentistry “Scuola Medica Salernitana” University of Salerno Baronissi Italy; ^5^ Department of Hematology‐Oncology Azienda Universitaria Ospedaliera Renato Dulbecco Catanzaro Italy; ^6^ Hematology and Transplant Center Ospedale Vito Fazzi Lecce Italy; ^7^ Hematology Hospital ‘Antonio Cardarelli’ Naples Italy; ^8^ Hematology and Transplant Center AOU Policlinico Bari Italy; ^9^ Hematology Unit Azienda Ospedaliera di Cosenza Cosenza Italy; ^10^ Hematology Unit IRCCS Giovanni Paolo II Bari Italy; ^11^ Hematology and Transplant Center Hospital “Monsignor Dimiccoli” Barletta Italy; ^12^ Hematology Hospital ‘San Giuseppe Moscati’ Avellino Italy; ^13^ Hospital Sant'Anna e San Sebastiano Caserta Italy

**Keywords:** momelotinib, myelofibrosis, myeloproliferative neoplasms, real‐life

## Abstract

Momelotinib, a novel JAK1/2 inhibitor with inhibitory activities on activin A receptor type I, has shown breakthrough clinical efficacy in patients with myelofibrosis (MF) and anemia, a disease‐related manifestation of challenging management. In this retrospective real‐life multicenter Italian study, we investigated the safety and efficacy of momelotinib in a cohort of 39 consecutive MF patients, regardless of prior therapy. The median duration of treatment was 7 months, and the overall response rate was 56% in transfusion‐dependent patients and 46% in the transfusion‐independent group. At 24 weeks of treatment, a hemoglobin increase > 1.5 g/dL was observed in 26% of patients, and constitutional symptom improvement was reported in 51% of cases, with a spleen volume reduction > 35% in 28%. Therapy discontinuation occurred in 18% of patients, with only one leukemia progression and three deaths during follow‐up. The safety profile was similar to that reported in clinical trials, with most toxicities of grade I‐II. In conclusion, our real‐life results support the use of momelotinib as an effective and safe therapeutic option for heavily pre‐treated, cytopenic MF patients in real‐world clinical practice.

## 1 Introduction

Myelofibrosis (MF), a clonal Philadelphia chromosome‐negative myeloproliferative neoplasm (MPN), is characterized by progressive bone marrow (BM) fibrosis, leading to extramedullary hematopoiesis, peripheral cytopenias, significant splenomegaly, and increased risk of acute myeloid leukemia (AML) development [1, 2, 3, 4]. Somatic driver mutations in the *JAK2* gene are common, while mutations in *CALR* or *MPL* genes are less frequently observed, and 8%–10% of patients lacking either of these mutations are classified as triple‐negative [5, 6]. Pharmacologic inhibition of the JAK–STAT pathway remains the only available and effective therapeutic choice for treatment of MF in older patients, and anti‐JAK1/2 agents, such as ruxolitinib and fedratinib, can provide symptom relief, splenic volume reduction, and improvement of survival [7, 8, 9, 10, 11, 12]. However, JAK inhibition can frequently exacerbate anemia, leading to transfusion dependency, and its management remains an unmet clinical need.

Dysregulated hepcidin signaling plays a pivotal role in MF, as its elevation impairs iron metabolism, ultimately suppressing erythropoiesis [13, 14, 15]. Momelotinib, a novel JAK1/2 inhibitor, also targets ACVR1, a key regulator of hepcidin expression [16]. This dual mechanism supports its unique potential to alleviate anemia while retaining JAK inhibition. In September 2023, the FDA approved momelotinib for patients with intermediate‐ or high‐risk MF and anemia, based on results from the MOMENTUM phase 3 trial, regardless of prior therapy [17]. In Italy, momelotinib is part of routine clinical practice only from May 2025; therefore, real‐life experiences remain limited. In this multicenter observational Italian study, we evaluated the efficacy and safety of momelotinib in patients with primary or secondary MF and anemia, previously treated with JAK inhibitors.

## 2 Patients and Methods

A total of 39 consecutive patients were enrolled from 12 Italian Hematology Units between March 2022 and April 2024. All patients started momelotinib through Expanded Access Programs (EAPs), outside of clinical trials. Eligible patients were aged ≥ 18 years and had a confirmed diagnosis of primary or secondary MF, according to the 2022 World Health Organization (WHO) classification [18]. This study was conducted in accordance with the Declaration of Helsinki, the International Conference on Harmonization Good Clinical Practice guidelines [19], and protocols approved by our Ethics Committee “Campania Sud,” Brusciano, Naples, Italy (prot./SCCE no. 24988). Written informed consent was obtained from all participants.

The primary endpoint was improvement in anemia, assessed according to the revised International Working Group–European LeukemiaNet (IWG‐ELN) criteria for MF [20]. Transfusion dependency was defined as having received ≥ 3 units of red blood cells (RBCs) within 12 weeks prior to momelotinib initiation, and in this patient, a major response was defined as achieving transfusion independence for 12 consecutive weeks associated with an average hemoglobin increase of ≥ 1.5 g/dL. Conversely, a minor response was defined as a > 50% reduction in transfusion requirements. In patients who were not transfusion dependent at baseline, a major response was defined as an average hemoglobin increase of ≥ 1.5 g/dL over 12 weeks, while a minor response required an increase of ≥ 1.0 g/dL.

Secondary endpoints included: spleen longitudinal diameter reduction; safety, assessed according to the Common Terminology Criteria for Adverse Events (CTCAE) v6; overall survival (OS); time on treatment (ToT); rate of AML transformation; improvement of MF‐related constitutional symptoms; and rate of hemoglobin level increase > 1.5 or 1 g/dL at 24 weeks.

### 2.1 Statistical Analysis

Data were collected in spreadsheets and analyzed using R statistical software (v. 4.0.5; RStudio) and SPSS (v. 25; IBM). Differences between groups were assessed by Chi‐square, Fisher's, Wilcoxon signed‐rank, or unpaired two‐tailed *t*‐tests. Kaplan–Meyer, log‐rank, and Breslow tests were used for survival analysis, and univariate and multivariate Cox regression models were used to examine effects (odd ratio, OR) of independent variables on outcomes. Paired boxplot analysis was employed to assess changes in spleen size before and after momelotinib treatment. A swimmer plot analysis was conducted to visualize the duration of therapy at the individual level, stratified by clinically relevant variables. All statistical tests were two‐sided, and a *p* value < 0.05 was considered statistically significant.

## 3 Results

### 3.1 Patients' Characteristics at Baseline

A total of 39 consecutive MF patients were included in this study (Table S1). The median follow‐up was 8 months (95% CI, 6–11 months), and the median age at momelotinib initiation was 71 years (range, 5–85 years). Most patients received a diagnosis of primary MF (72%), while 28% had secondary MF, with a median time to progression from a previous MPN of 138 months (range, 29–370 months). Splenomegaly was present in 92% of patients, with a median spleen longitudinal diameter of 19 cm (range, 11.5–27.5 cm). Median baseline hemoglobin levels were 8.7 g/dL (range, 6.9–15.8 g/dL), with 41% of patients being transfusion‐dependent and 33% of them with a high transfusion burden.

In our cohort, 84% of patients were previously treated with ruxolitinib, predominantly as first‐line therapy (79%), with a median exposure of 18.5 months (range, 1–87 months). Anemia worsening during ruxolitinib was reported in 58% of cases, and 64% were treated with concomitant subcutaneous erythropoietin. Ruxolitinib was discontinued in 56% of cases due to treatment failure, in 26% due to hematological toxicity, and in 2% of cases for the patient's choice. Fourteen patients (36%) received fedratinib, mainly as second‐line therapy, with a median exposure of 10.5 months (range, 2–22 months), and anemia worsening and concomitant use of erythropoietin were reported in 31% and 33% of cases, respectively.

### 3.2 Real‐World Efficacy of Momelotinib

**FIGURE 1:**
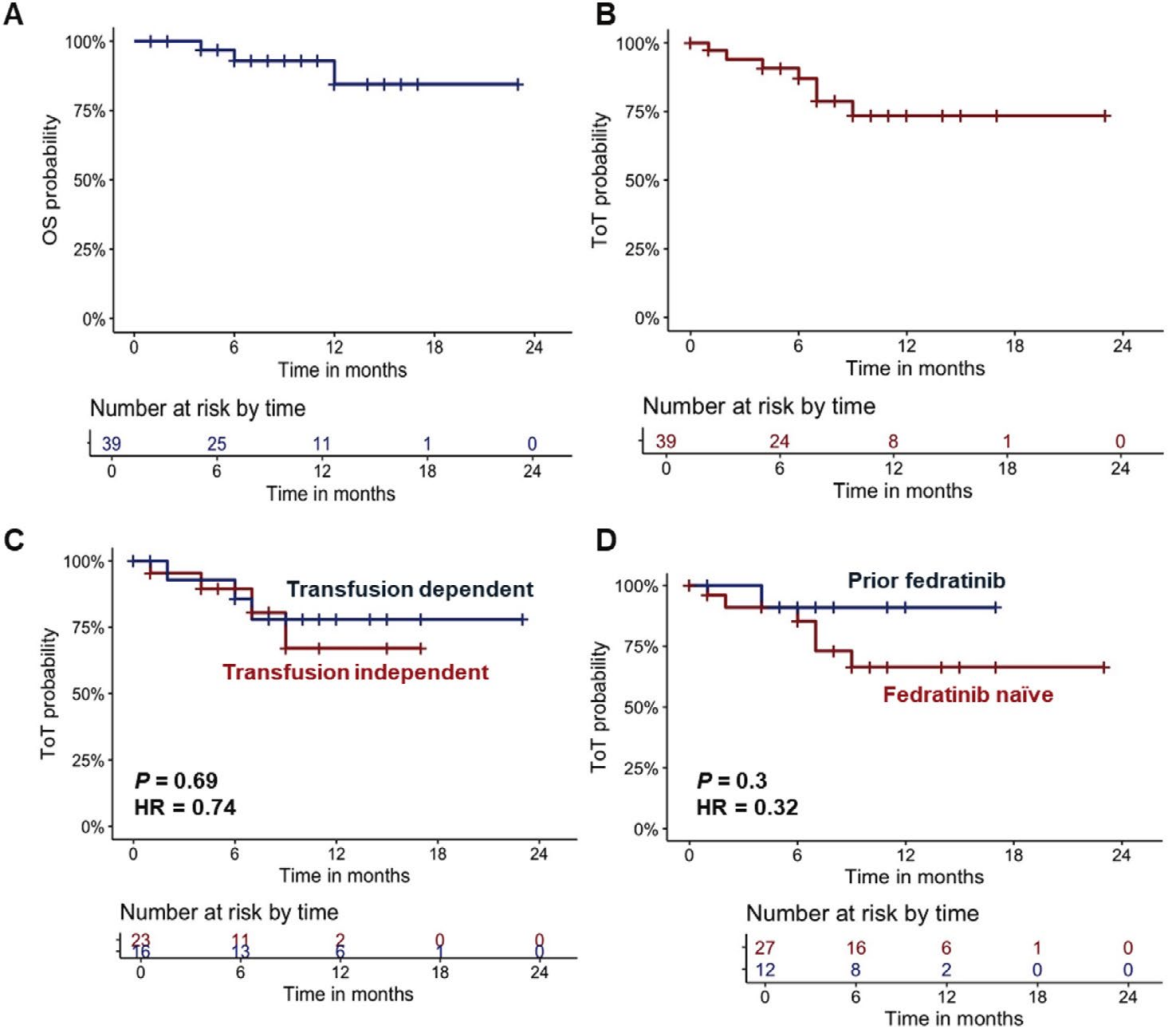
Clinical outcomes of patients receiving momelotinib. (A) Overall survival (OS) and (B) Time on treatment (ToT) of patients with myelofibrosis and anemia treated with momelotinib (*N* = 39). Patients were also stratified by transfusion dependency (C) or prior administration of another JAK inhibitor, fedratinib (D), and ToT is reported. The number of censored subjects at risk is shown. HR, hazard ratio.

At data cut, the median time from MF diagnosis to momelotinib initiation was 34 months (range, 2–291 months), and the median duration of momelotinib therapy was 7 months (range, 0–23 months) (Table 1). Overall response rate (ORR) in transfusion‐dependent patients was 56% with 37% achieving a major response and 19% a minor response; in those with severe transfusion dependency, ORR was 54% (31% major and 23% minor response). In transfusion‐independent patients, ORR was 46% (27% major and 19% minor response). The median time to achieve transfusion independence was 55 days (range, 31–360 days). At 24 weeks of treatment, hemoglobin increase > 1.5 g/dL was observed in 26% of patients, and > 1 g/dL in 10%. Constitutional symptom improvement was reported in 51% of cases, and spleen volume reduction > 35% in 28%. Therapy discontinuation occurred in 18% of patients, due to hematological toxicity (2%), death (2%), lack of response (5%), other neoplasms (2%), or unknown reasons (2%). Median OS was not reached, with a 1‐year OS of 85% (95% CI, 68–100%). Only one AML progression (2%) and three deaths during follow‐up (8%) were observed (Figure 1A). The median ToT was also not reached, with 74% of patients estimated to remain on therapy at 24 months (Figure 1B). ToT was not significantly influenced by baseline variables, including transfusion dependency status (Figure 1C,D).

**TABLE 1:** Clinical outcomes during momelotinib treatment.

Outcomes	Entire cohort *N* = 39
Median time to momelotinib initiation, months (range)	34 (2–291)
Median duration of treatment, months (range)	7 (0–23)
Median time on treatment, months (95% CI)	NR (NR‐NR)
Therapy discontinuation, *n* (%)	7 (18)
Reasons for therapy discontinuation, *n* (%)	
Hematological toxicity	2 (2)
Death	1 (2)
No response	2 (5)
Other neoplasia	1 (2)
Not available	1 (2)
ORR of transfusion‐dependent patients, *n* (%)	9 (56)
Major response	6 (37)
Minor response	3 (19)
ORR of severe transfusion‐dependent patients, *n* (%)	7 (54)
Major response	4 (31)
Minor response	3 (23)
ORR of transfusion independent patients, *n* (%)	12 (46)
Major response	7 (27)
Minor response	5 (19)
Median time to transfusion independency, days, (range)	55 (31–360)
Hb increase > 1.5 g/dL at 24 weeks, *n* (%)	13 (33)
Hb increase > 1 g/dL at 24 weeks, *n* (%)	18 (46)
Constitutional symptom improvement, *n* (%)	(51)
Cardiovascular events during momelotinib treatment, *n* (%)	1 (2)
AML progression, *n* (%)	1 (2)
Deaths, *n* (%)	3 (8)
OS, median, months (range)	NR (1–23)
12‐month OS, %, (95% CI)	85 (68–100)

Abbreviations: AML, acute myeloid leukemia; CI, confidential interval; Hb, hemoglobin; ORR, overall response rate; OS, overall survival.

**FIGURE 2:**
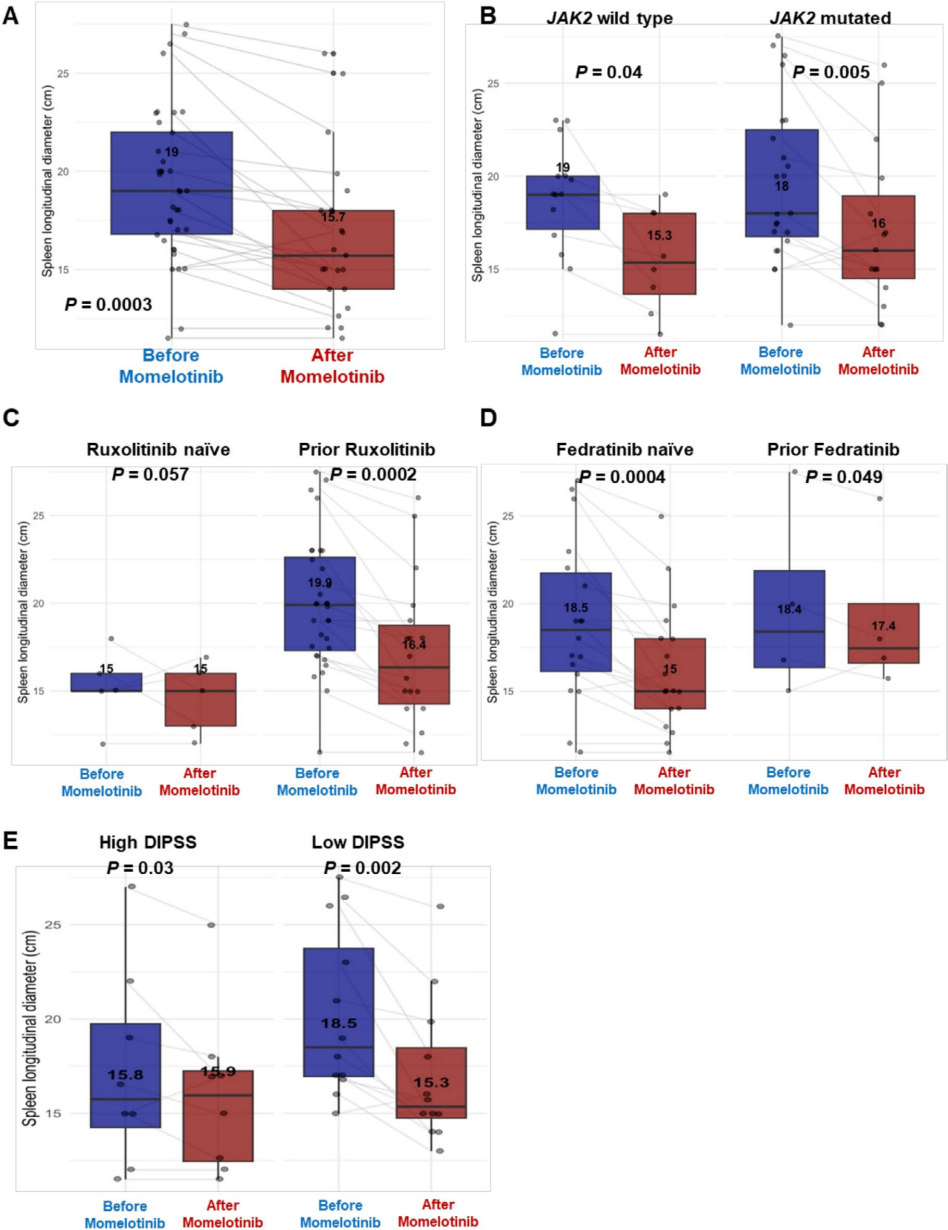
Spleen reduction in patients treated with momelotinib. Spleen longitudinal diameter variations are reported for (A) the entire cohort (*N* = 39) before and after momelotinib administration, or divided based on (B) the presence of *JAK2* mutation, prior (C) ruxolitinib or (D) fedratinib treatment, and (E) Dynamic International Prognostic Scoring System (DIPSS) score.

**FIGURE 3:**
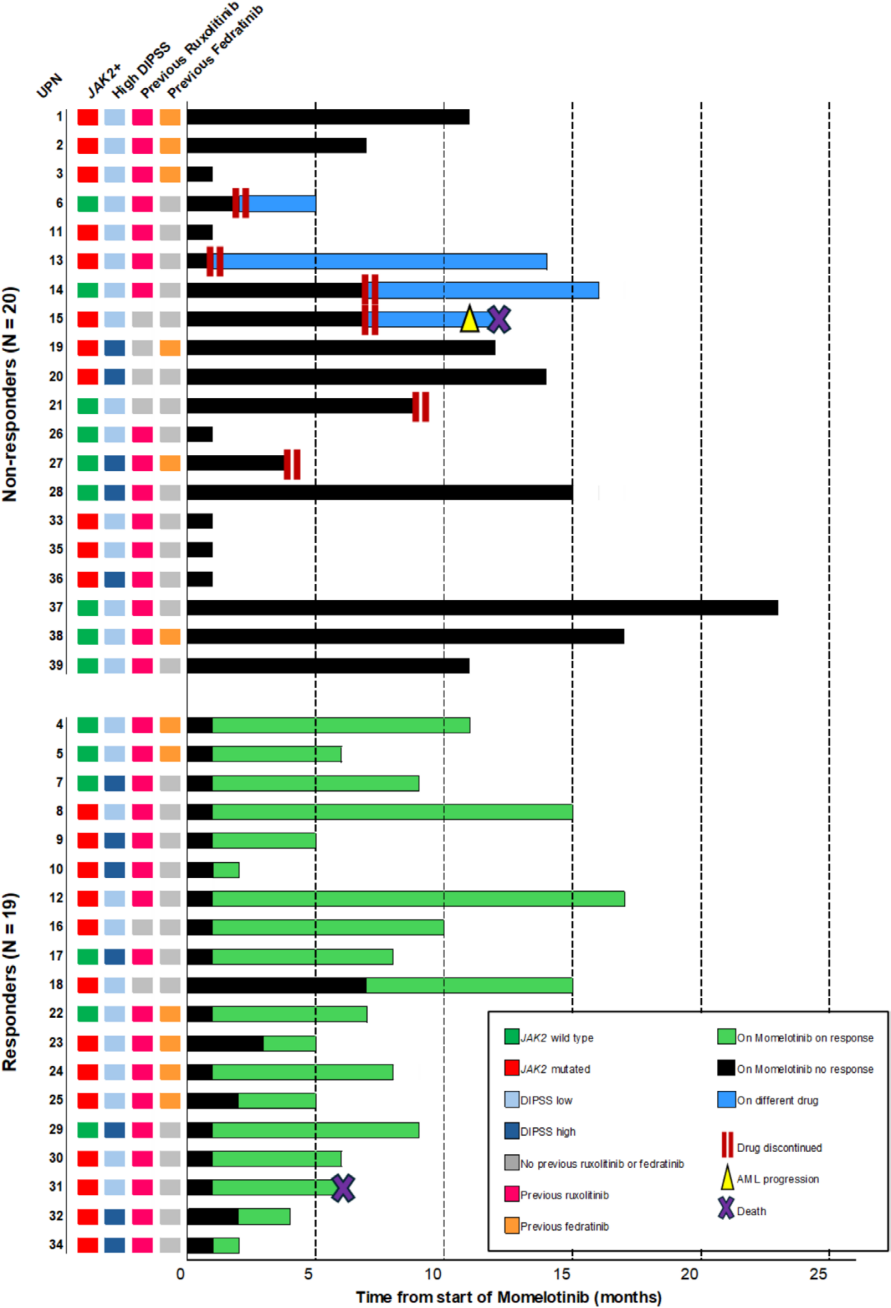
Clinical outcomes of the 39 patients with myelofibrosis and anemia enrolled in the study. Swimmer plot with the clinical outcomes of responders (*N* = 19) and non‐responders (*N* = 20). Bars represent the follow‐up time for each patient. On the timeline, black bars represent the start of momelotinib treatment until the primary outcome (anemia improvement); green bars represent the time for which patients continued momelotinib on clinical responses, while blue bars represent the time that patients discontinued momelotinib (double vertical bars) and switched to a different drug. Yellow triangles indicate the time of acute myeloid leukemia (AML) progression, and purple crosses indicate that the patient died.

A significant decrease in the median spleen longitudinal diameter was documented (from 19.0 cm at baseline to 15.7 cm post‐treatment; *p* = 0.0003) (Figure 2A). This reduction was observed in all patients' categories, except for high‐risk DIPSS subjects and those previously treated with fedratinib (Figure 2B–E). The swimmer plot illustrates the duration of momelotinib therapy at the individual level, with hematological response commonly observed within the first 3 months of therapy. Late response (> 5 months of treatment) was registered only in one case (Figure 3).

### 3.3 Safety

Momelotinib was generally well tolerated (Table 2). Reported adverse events included thrombocytopenia (7%), pneumonia (5%), non‐ST elevation myocardial infarction (2%), cardiac arrest (2%), transaminase elevation (5%), renal dysfunction (5%), diarrhea (5%), and peripheral neuropathy (2%). Most toxicities were grade I–II.

**TABLE 2:** Safety.

Toxicity	Entire cohort *N* = 39
Thrombocytopenia, *n* (%)	
Grade I–II	1 (2)
Grade III–IV	2 (5)
Pneumonia, *n* (%)	
Grade I–II	2 (5)
Grade III–IV	—
Non‐ST‐elevation myocardial infarction, *n* (%)	1 (2)
Cardiac arrest, *n* (%)	1 (2)
Hypertransaminasemia, *n* (%)	
Grade I–II	—
Grade III–IV	2 (5)
Renal failure, *n* (%)	
Grade I–II	2 (5)
Grade III–IV	—
Diarrhea, *n* (%)	
Grade I–II	—
Grade III–IV	2 (5)
Peripheral neuropathy, *n* (%)	
Grade I–II	1 (2)
Grade III–IV	—

### 3.4 Exploratory Analyses

No predictive factors of responsiveness to momelotinib were identified in univariate logistic regression, although a trend toward lower response was observed in patients with higher baseline hemoglobin levels (OR, 0.69; *p* = 0.075) (Table S2). No associations were found with LDH or ferritin levels, transfusion dependency, *JAK2* mutational status, MF subtype, DIPSS score, age, or prior exposure to JAK inhibitors.

## 4 Discussion

In this real‐life observational multicenter Italian study, we evaluated the efficacy and safety of momelotinib in patients with primary or secondary MF and anemia, and we confirm its role as a disease‐modifying agent with a unique dual mechanism: JAK1/2 inhibition and suppression of hepcidin via ACVR1 blockade. According to the updated criteria for evaluating MF‐related anemia response [20], the ORR in transfusion‐dependent patients reached 56%, with a major response observed in 37% of cases, consistent with results from the phase III MOMENTUM trial [17]. Notably, ORR remained robust (54%) also in our severely transfusion‐dependent patients, despite their clinical heterogeneity and the absence of strict inclusion criteria. Moreover, a hemoglobin improvement > 1.5 g/dL at 24 weeks was documented in 33% of patients, further supporting the anti‐hepcidin effect of momelotinib in restoring effective erythropoiesis.

Momelotinib has been initially considered less potent than other JAK inhibitors for controlling splenomegaly; however, additional evidence shows non‐inferiority compared to ruxolitinib in spleen volume reduction [21]. In our cohort, we observed a significant reduction in spleen size, with a median spleen longitudinal diameter decrease from 19.0 cm at baseline to 15.7 cm during momelotinib treatment, especially in patients with lower DIPSS scores and those fedratinib‐naïve. In contrast to the SIMPLIFY‐1 trial results, a consistent improvement in constitutional symptoms and quality of life was observed in 51% of our patients, reinforcing momelotinib's therapeutic potential in routine clinical practice [21].

The safety profile of momelotinib was favorable and manageable, as adverse events were more frequently low‐grade, while serious cardiovascular events (2% non‐ST elevation myocardial infarction, 2% cardiac arrest) and grade III–IV hematologic toxicity were uncommon. These results were similar to those observed in the MOMENTUM trial, suggesting that momelotinib can be safely administered in real‐world settings, even in elderly patients with comorbidities.

Our study has several limitations: (i) the retrospective nature of data collection; (ii) the short median follow‐up (8 months); (iii) mutational data beyond driver mutations not systematically available; (iv) spleen response was assessed using longitudinal diameter rather than volumetric measurements; and (v) limited sample size and lack of a control group. Additionally, treatment practices could vary across centers, potentially introducing heterogeneity in patient management and response assessment.

Despite these limitations, the clinical benefits of momelotinib were clearly observed in our study, which represents one of the first real‐world evaluations of momelotinib outside clinical trials, offering valuable insights into its utility in routine clinical practice. Momelotinib has demonstrated substantial clinical activity in an unselected, real‐world population of patients with MF and anemia, including those previously treated with other JAK inhibitors. In our cohort, a higher spleen response tended to be observed in patients with lower‐risk disease (DIPSS < 3), suggesting that earlier use of momelotinib could enhance clinical benefit. However, prospective studies with longer follow‐up and comprehensive molecular profiling are warranted to identify predictive markers of response, validate clinical predictors, and optimize patient selection for momelotinib therapy.

## Author Contributions

Conceptualization: C.S. Data collection: M.C.M., N.P., M.D.P., D.D.N., A.L., L.D.F., G.L., I.P., M.M., Ca.Co., A.R., A.B., F.M., V.D.F., T.M.S., R.B., G.M., R.F., R.G., B.S., S.L., A.M.D.C., A.M., V.G., F.F., G.T., A.G., M.G., P.M., M.A., N.D.R., M.R., Ca.Ca., and F.P. Methodology: D.D.N., A.M.D.C., and V.G. Clinical data: M.C.M., N.P., M.D.P., D.D.N., A.L., L.D.F., G.L., I.P., M.M., Ca.Co., A.R., A.B., F.M., V.D.F., T.M.S., R.B., G.M., R.F., R.G., B.S., S.L., A.M.D.C., A.M., V.G., F.F., G.T., A.G., M.G., P.M., M.A., N.D.R., M.R., Ca.Ca., and F.P. Data analysis: D.D.N. and V.G. Writing – original draft preparation: M.C.M., D.D.N., and V.G. Writing – review and editing: F.F., G.T., A.G., M.G., P.M., M.A., N.D.R., M.R., Ca.Ca., F.P., and C.S. All authors have read and agreed to the published version of the manuscript.

## Disclosure

The authors declare that the material is original, has not been published before, nor is under consideration in any journal.

## Consent

Patients received informed consent obtained in accordance with the Declaration of Helsinki (World Medical Association 2013) and protocols approved by the local ethics committee (Ethics Committee “Campania Sud”, Brusciano, Naples, Italy; prot./SCCE n. 24 988).

## Conflicts of Interest

The authors declare no conflicts of interest.

## Supporting information


**Table S1:** Clinical characteristics at baseline.
**Table S2:** Univariate logistic regression on probability to achieve hematological response during momelotinib treatment.

## Data Availability

The data that support the findings of this study are available from the corresponding author upon reasonable request.
