# Supplementary material for: Efficacy of Momelotinib in Myelofibrosis Patients: Results From a Multicenter Study
Source: Eur J Haematol. 2025 Sep 16;116(1):23–30. doi: 10.1111/ejh.70034 (PMC12673357; doi:10.1111/ejh.70034)
Supplement: Supplementary file 1 — Table S1: Clinical characteristics at baseline. Table S2: Univariate logistic regression on probability to achieve hematological response during momelotinib treatment. [file EJH-116-23-s001.docx]

**Supplementary Table 1. Clinical characteristics at baseline.**

| **Characteristics** | **Entire cohort**  **N = 39** |
| --- | --- |
| **Median age, years (range)**  **Male, n (%)** | 71 (5-85)  25 (64) |
| **Diagnosis, n (%)**  Primary Myelofibrosis  Secondary Myelofibrosis  PV  ET  ET+CML | 28 (72)  11 (28)  7 (17)  3 (7)  1 (2) |
| **Median time of disease progression, months (range)** | 138 (29-370) |
| **Driver mutations, n (%)**  *JAK2*  *CALR*  *MPL*  Triple negative | 24 (61)  6 (15)  3 (7)  6 (15) |
| **Other mutations, n (%)**  *TET2*  *EZH2*  *SETBP1*  *U2AF1*  *KRAS*  *ASXL1*  *SF3B1* | 1 (2)  1 (2)  1 (2)  1 (2)  1 (2)  2 (5)  2 (5) |
| **DIPSS score, n (%)**  Low  Intermediate-1  Intermediate-2  High  Not available | 0  8 (21)  16 (41)  12 (31)  3 (7) |
| **Splenomegaly, n (%)** | 36 (92) |
| **Hepatomegaly, n (%)** | 15 (38) |
| **Median spleen longitudinal diameter, cm (range)** | 19 (11.5-27.5) |
| **LDH, median, U/L (range)** | 468 (200-2426) |
| **Median hemoglobin, gr/dl, (range)**  **Transfusion dependency*, n (%)**  **Severe transfusion dependency^§^, n (%)** | 8.7 (6.9-15.8)  16 (41)  13 (33) |
| **Comorbidities, n (%)**  Prior Vein Thrombosis  Diabetes  Hypertension  Stroke or TIA  Non-hematological cancer  HBcAB positivity | 9 (23)  5 (13)  17 (44)  2 (5)  8 (21)  6 (15) |
| **Prior ruxolitinib, n (%)**  First line  Second line | 33 (84)  31 (79)  2 (5) |
| **Median Ruxolitinib exposure, months (range)** | 18.5 (1-87) |
| **Concomitant use of erythropoietin, n (%)** | 25 (64) |
| **Anemia worsening during ruxolitinib, n (%)** | 23 (58) |
| **Reasons of ruxolitinib discontinuation, n (%)**  Treatment failure  Hematological toxicity  Patient’s choice | 22 (56)  10 (26)  1 (2) |
| **Prior fedratinib, n (%)**  First line  Second line  Third line | 14 (36)  1 (2)  12 (31)  1 (2) |
| **Median Fedratinib exposure, months (range)** | 10.5 (2-22) |
| **Concomitant use of erythropoietin, n (%)** | 13 (33) |
| **Anemia worsening during fedratinib, n (%)** | 12 (31) |
| **Reasons of fedratinib discontinuation, n (%)**  Treatment failure  Hematological toxicity | 4 (10)  10 (26) |

**Abbreviations.** PV, polycythemia vera; ET, Essential thrombocythemia; CML, chronic myeloid leukemia; DIPSS, Dynamic International Prognostic Scoring System; TIA, transient ischemic attack; HBcAB, Hepatitis B Core antibody. * ≥ 3 red blood cell units in 12 weeks; § ≥ 6 red blood cell units in 12 weeks.

**Supplementary Table 2. Univariate logistic regression on probability to achieve hematological response during momelotinib treatment.**

|  | **OR** | **95%CI** | **P value** |
| --- | --- | --- | --- |
| **Hemoglobin** | 0.69 | 0.44-1 | 0.075 |
| **LDH** | 1 | 1 | 0.81 |
| **Ferritin** | 1 | 1 | 0.67 |
| **Transfusion dependency** | 1.09 | 0.3-3.97 | 0.89 |
| ***JAK2* mutation** | 1.77 | 0.48-6.83 | 0.39 |
| **Secondary myelofibrosis** | 1.38 | 0.34-5.85 | 0.65 |
| **High transfusion burden** | 1.17 | 0.29-4.69 | 0.82 |
| **DIPSS = 3** | 1.4 | 0.35-5.94 | 0.63 |
| **Age** | 1.02 | 0.98-1.08 | 0.42 |
| **Prior ruxolitinib** | 2.12 | 0.36-16.9 | 0.41 |
| **Prior fedratinib** | 1.08 | 0.27-4.28 | 0.91 |

Abbreviations. OR, odds ratio; CI, confidential interval; LDH, lactate dehydrogenase; DIPSS, Dynamic International Prognostic Scoring System.
